# Assessing receptive verb knowledge in late talkers and autistic children: Advances and cautionary tales

**DOI:** 10.21203/rs.3.rs-2613423/v1

**Published:** 2023-03-02

**Authors:** Sabrina Horvath, Sudha Arunachalam

**Affiliations:** 1 Medical University of South Carolina; 2 New York University

**Keywords:** late talkers, autism spectrum disorder, vocabulary, verbs, assessment, eye-tracking

## Abstract

**Purpose:**

Using eye-tracking, we assessed the receptive verb vocabularies of late talkers and typically developing children (Experiment 1) and autistic preschoolers (Experiment 2). We evaluated how many verbs participants knew and how quickly they processed the linguistic prompt.

**Method:**

Participants previewed two dynamic scenes side-by-side (e.g., “stretching” and “clapping”) and were then prompted to find the target verb. Children’s eye gaze behaviors were operationalized using established approaches in the field with modifications in consideration for the type of stimuli (dynamic scenes versus static images) and the populations included. Accuracy was calculated as a proportion of time spent looking to the target, and linguistic processing was operationalized as latency of children’s first look to the target.

**Results:**

In Experiment 1, there were no group differences in the proportion of verbs known, but late talkers required longer to demonstrate their knowledge than typically developing children. Latency was predicted by age but not language abilities. In Experiment 2, autistic children’s accuracy and latency were both predicted by receptive language abilities.

**Conclusion:**

Eye gaze can be used to assess receptive verb vocabulary in a variety of populations, but in operationalizing gaze behavior, we must account for between- and within-group differences. Bootstrapped cluster-permutation analysis is one way to create individualized measures of children’s gaze behavior, but more research is warranted using an individual differences approach with this type of analysis. Finally, latency may not be a valid measure for dynamic scene stimuli for children under three years old.

## Introduction

Advances in eye-tracking technology and research highlight a tantalizing prospect: It may be possible to develop a standardized vocabulary assessment that relies on eye-tracking. Such an assessment would be of benefit for evaluating language knowledge in populations who have difficulty with pointing or vocalizing to indicate their knowledge, including children with motor impairments such as cerebral palsy (1–2), autistic children (3–5), or very young children (6–7). Children in these populations are known to struggle with standardized assessments because of the behavioral demands of the task, oftentimes being unable to participate (e.g., 4–5). In this way, an eye-tracking assessment is an ideal solution to a major clinical need.

In this paper, we focus specifically on using eye-tracking to assess receptive vocabulary. Our results offer advances toward the development of eye-tracking vocabulary assessment but also highlight gaps in our knowledge. While such an assessment is far from ready for clinical application—limited in part by cost for clinicians—addressing the gaps we have identified is a necessary step toward this long-term goal.

Dozens of studies have demonstrated the feasibility of using eye-tracking to assess children’s receptive vocabulary (e.g., 4, 8–11). Broadly, these studies follow the same experimental paradigm. Each trial begins with a “Baseline Phase” where children preview multiple potential candidate scenes (e.g., pictures of a ball and a shoe). In the “Prompt Phase,” children are directed to look to one of the scenes (e.g., “Where’s the ball?”). The time after this linguistic prompt is the “Test Phase” of the trial, from which children’s eye-gaze behavior is analyzed to determine knowledge.

Most of these studies have focused only on one type of vocabulary word: nouns labeling objects or animals. However, a comprehensive assessment should include many types of words. We argue that verbs are critically important to consider. Verb meanings are strongly tied to the structure of the sentences in which they appear, and verb knowledge is a better predictor of later grammatical outcomes than noun knowledge (12). Therefore, studying children’s verb knowledge may be particularly revealing for understanding the diversity in language outcomes as well as for the early assessment of language disorders.

However, in the context of eye-tracking assessments, testing verb knowledge raises unique challenges. Prior studies using eye gaze to assess noun knowledge have primarily used static images such as a picture of a ball. Because most early-acquired verbs denote dynamic events that unfold over time, static images are not good depictions; consider, for example, the difficulty of distinguishing “catching” and “throwing” with a single static image. Although some standardized assessments such as the Peabody Picture Vocabulary Test (13) depict verb referents using static images, these are not ideal for young children, who have difficulty interpreting the symbols used to denote dynamic action (e.g., lines to indicate motion) and extrapolating movement from them (14–15).

Instead, videos of dynamic scenes better illustrate verb meanings. However, using dynamic scenes requires rethinking how eye gaze measures are operationalized because gaze behaviors differ when viewing dynamic scenes as compared to static images (e.g., 9). Few studies have used dynamic scenes to depict verbs, and among those that have, there is not a consensus on what eye gaze measures are best nor how they should be operationalized (6, 9–11, 16–18).

Additionally, much of the research on eye-gaze vocabulary assessments has included only typically developing children. However, in developing assessments for children with language and communication disorders, we must consider the possibility that their performance differs substantially from their typically developing peers’. For example, late talkers, children with developmental language disorder, and autistic children are all slower than typically developing children to process language (19–22), meaning that they may take longer to settle their gaze on the scene depicting the correct referent.

It is critical that researchers understand how to adapt receptive vocabulary tasks for children with differing abilities. For example, Brady et al. (3) have argued that because pointing is challenging for autistic children, using eye gaze will more appropriately capture their vocabulary knowledge if we can reliably interpret their gaze behaviors. Several studies have explored the possibility of assessing autistic children’s vocabulary using eye gaze (e.g., 3–4, 23–28), but they have focused almost exclusively on noun vocabulary and static image targets.

In this study, we address these two gaps. Our goal is to advance understanding of the potential for eye-gaze vocabulary assessments. First, we focus specifically on verbs, depicting verb referents as dynamic scenes rather than static images. Second, we include children with language delays and disorders: specifically, late talkers and autistic children. Prior research indicates that both populations differ in their eye-gaze behaviors during receptive vocabulary tasks as compared to typically developing children, at least given noun trials and static images (3–4, 20, 23, 28).

Approximately 15% of two-year-olds are late talkers, defined by having atypically small expressive vocabularies for their age with no known cognitive or developmental disorders (29). Late talkers are at increased risk for developmental language disorder (30–32). Many late talkers have receptive language delays as well, but others do not (33–34). Importantly, there appear to be differences in how late talkers build their vocabularies (35–38), including specifically their verb vocabularies (39–40).

Autistic children have notoriously heterogeneous language abilities (21–22; 41–43), ranging from non-verbal to no expressive or receptive language deficits. However, most autistic children have below-age language abilities, including in vocabulary knowledge (e.g., 43–44). Receptive language abilities vary widely, but for many, receptive language is even more impaired than expressive language (e.g., 41). Further, autistic children have particular difficulty participating in standardized assessments which require overt behavioral responses (3–5) making alternative assessment methodologies an important clinical need.

### Operationalizing eye gaze behaviors

Given the richness of the dataset collected during eye-tracking, interpreting gaze behavior to measure vocabulary knowledge is a daunting task. Prior studies have converged on two distinct measures: accuracy and processing speed. Because this is a methods study, we will discuss each measure below in detail, including how it has been operationalized in prior research and how it may need to be modified for 1) verb trials with dynamic scenes and 2) children with language delays and disorders. In so doing, we recognize that “children with language delays and disorders” is not a monolith, and that there is considerable variability across and within disorder profiles. To this point, methods must be adapted to specific populations and even, potentially, to individuals.

#### Accuracy

Accuracy—or, whether a child knows the target word—is operationalized as a proportion of looking time to the target scene. There are two common ways of calculating accuracy. One approach (8,20) is to compare the proportion of time spent looking to the target versus distractor scenes during the Test Phase. If the child prefers the target to the distractor, they are credited with knowing the target word. An alternative approach (7, 45–46) is to compare the proportion of time spent looking to the target scene before versus after the linguistic prompt is provided (i.e., Baseline versus Test). If this proportion looking increases by a predetermined threshold, typically 15%, then the child is credited with knowing the target word (7). We use this second method because it accounts for idiosyncratic preferences that children may have for one scene over the other. This second approach has been used with static images and noun targets (45–46) as well as dynamic scenes and verb targets (9, 11). Further, accuracy scores derived from this approach correlate with concurrent vocabulary knowledge (9, 11).

Regardless of the approach, researchers must identify a time window within the Test Phase from which to calculate accuracy. Children’s gaze is predicably drawn to the target immediately after the linguistic prompt, but afterward, they look around to other locations on the screen. Fernald and colleagues (8, 20, 47–48), working with typically developing toddlers viewing noun trials with static images, use a time window for accuracy calculations of 300 to 1800 msec after the linguistic prompt. Children require 300 msec to coordinate and launch an eye movement toward the target, and their attention patterns are less consistent after 1.5 s of gaze. But, what about children who are not typically developing, or for verb trials with dynamic scene stimuli?

Valleau and colleagues (11) have observed that this 300 to 1800 msec window is likely inappropriate for verb trials with dynamic scenes. In a study including typically developing 22-to 24-month-old toddlers, Valleau et al. found that, on noun trials, toddlers showed evidence of preferring the target scene within 300 to 1800 ms after the target noun was queried, consistent with Fernald and colleagues’ research. However, on verb trials, the same toddlers required additional time to orient their gaze. It remains an open question in research as to what the window should be used to calculate accuracy given verb targets and dynamic scenes.

Furthermore, the appropriate time window for calculating accuracy may differ for typically developing children and those with language delays and disorders. For example, late talkers are slower language processors than typically developing toddlers and may therefore take longer to settle on the correct scene (20). Autistic children are also slower language processors than typically developing peers (21–22) and engage in different patterns of gaze behavior, including when viewing social stimuli (4, 49). We therefore expect that both late talkers and autistic children require more time than typically developing children to demonstrate knowledge, and that there may be considerable within-group variability, particularly for autistic children.

Given that there is no *a priori* hypothesis for when or how long the test window should be in accuracy calculations, we identify the appropriate windows using a bootstrapped cluster-based permutation analysis (50). Bootstrapped cluster-based permutation analysis is a systematic method for identifying when, and for how long, behavior differs between groups or conditions in studies that have time-locked data, such as eye-tracking or EEG studies. Small time windows are analyzed to determine whether there are differences in gaze behavior between conditions, and consecutive windows that show a difference are grouped together in clusters. Permutation testing is applied to derive a distribution of cluster values in order to determine whether the clusters are statistically significant. However, there are challenges in applying this approach to individual children’s performance, as we discuss below.

#### Processing speed

To evaluate processing speed, researchers typically measure children’s latency to the target image—that is, how long after the auditory prompt it takes children to first look at the target, irrespective of whether or when they shift gaze away afterward. Robust evidence from studies involving static images to depict noun targets has shown that this measure predicts concurrent language ability (8), concurrent noun-learning abilities (51) and later language and intelligence (20, 48, 52). Whether this measure can also be used with dynamic scene stimuli is less clear: If children happen to look at the distractor first, they may take longer than with static images to visually disengage from it. Indeed, Valleau et al. (11) found no association between latency and vocabulary size in typically developing children under two years of age for verbs and dynamic scenes. However, age may also be a factor: Koenig et al. (16) found that, for typically developing three-year-olds, latency correlated with vocabulary size on both noun and verb trials.

In considering children with language delays and disorders, latency has been shown to be a predictive measure given noun trials with static images. For example, Fernald and Marchman (20) demonstrated that late talkers have longer latencies than typically developing toddlers, and that late talkers’ latencies predicted vocabulary growth between 18 and 24 months. Autistic children, too, have slower language processing skills that correspond with other language measures (e.g., 53–56), including their accuracy on eye-tracking receptive vocabulary tasks (4). However, no studies have considered latency for children with language delays and disorders viewing dynamic scenes and verb stimuli.

### The present study

To begin to address these foundational gaps in our understanding of how eye gaze can be used to examine receptive verb vocabulary in children with language delays and disorders, we present two experiments that adapt tasks used by Valleau et al. (11) and Koenig et al. (16). Our goal is to explore how accuracy and processing speed measures may be adapted to accurately capture verb knowledge in children with language delays and disorders.

Experiment 1 includes late talkers and typically developing controls. Fernald and Marchman (20) found that, given noun trials and static images, late talkers knew fewer words and averaged slower latencies as compared to typically developing children. We therefore expect a receptive verb vocabulary task to reveal group-level differences between late talkers and typically developing children.

In Experiment 2, we ask how this same verb vocabulary task captures within-group variability among autistic children. Autistic children—including those in our sample—can present with a wider range of abilities, and therefore studying children on the autism spectrum allows us to examine associations and dissociations among language measures. We expect within-group variation commensurate with language abilities on other measures.

## Experiment 1: Late talkers and typically developing children

### Participants

The final sample included 45 children (17 female, 28 male) with an average age of 28.5 months (*SD* = 3.0 months, range = 24.5–34.7 months) recruited from the greater Boston area. The sample skewed male because we focused recruitment on late talkers, who are more likely to be male (31). Participants were prescreened for a history of hearing loss or tubes; additionally, children were screened at their visit for a high risk of autism spectrum disorder using the Modified Checklist for Autism in Toddlers, Revised (M-CHAT-R: 57). All participants in the final sample were classified as “low risk.” Per parent report, all children were exposed to English at least 70% of the time. Participants had no reported developmental disorders other than for language: Three children were reported to have a language-related diagnosis, either “expressive language delay” or “language delay.” Seven additional children participated but were excluded from final analysis due to a prolonged history of ear tubes (*n* = 2), a history of tongue-tie (*n* = 1), diagnosis with autism spectrum disorder within a week following participation (*n* = 1), or failure to complete the experimental session due to fussiness (*n* = 3).

Participants were primarily white (91%); 2% were Asian and 5% were more than one race. Nearly all children (96%) had at least one parent with a college degree or more advanced degree, including 35% of children who had at least one parent with a doctorate (Ph.D., M.D., or J.D.). One family did not provide information on race/ethnicity, and three families did not provide information on parent education.

Expressive vocabulary was assessed using the MacArthur-Bates Communicative Development Inventories Level 2 Short Form A (MBCDI: 58). Children were reported to produce, on average, 69 of the 100 words (*SD* = 27, range = 1 – 100). The Preschool Language Scales, 5^th^ edition (PLS: 59) was administered to characterize broader language abilities. Children averaged a standard score of 106 (*SD* = 17) on the Auditory Comprehension subscale (PLS-AC) and 107 (*SD* = 16) on the Expressive Communication subscale (PLS-EC). Finally, the Visual Reception subscale of the Mullen Scales of Early Learning (MSEL-VR: 60) was used as a proxy for nonverbal intelligence. Children had an average *T-*score of 53 (*SD* = 11); one participant did not complete MSEL-VR testing.

We used the MBCDI to classify each child as either a “late talker” (*n* = 14) or “typically developing” (*n* = 31). Of the late talkers, eight had a standard score at or below the 15^th^ percentile for their age and gender. The MBCDI is only normed for children up to age 30 months; however, children older than 30 months whose score was at or below the 15^th^ percentile for their gender at 30 months were also classified as late talkers (*n* = 4). An additional 2 late talkers were classified based on parent report that they had qualified for speech and language therapy because of late talking. In total, 10 late talkers had qualified for or were receiving speech therapy services at the time of participation; no typically developing children had any reported history of therapy. There were no group differences with respect to average age (*t* = 1.14, *p* = .26, *n.s*.), proportion male (*z* = 0.85, *p* = .39, *n.s*.), proportion monolingual English language learners (*z* = 1.5, *p* = .12, *n.s*.), or proportion who had at least one parent with a doctorate (*z* = 1.8, *p* = .06, *n.s*.).^1^ All standardized measures showed group differences. See Table 1.

### Apparatus

Stimuli were displayed on a 24-inch Tobii T60 XL corneal reflection eye-tracking monitor, which samples gaze approximately every 17 ms, calibrated at the beginning of each experimental session using a 5-point calibration procedure. Children sat in a car seat 20 inches from the monitor or in their parent’s lap while the parent wore a blindfold.

### Stimuli

**Figure 1.**
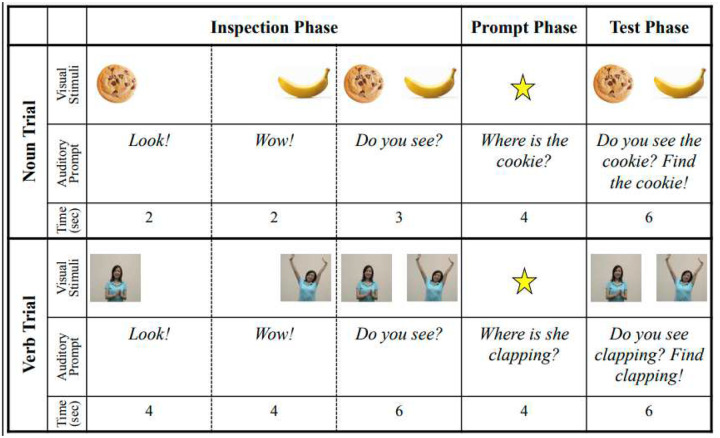
The trial structure of one trial for Experiments 1 and 2.

The stimuli were initially developed by Konishi et al. (61) and modified by Valleau et al. (11). Konishi et al. selected a total of 36 verbs and 14 nouns that are highly imageable and learned early in typical language development. They filmed 6-second video clips depicting the referent action for each verb and selected static images depicting the referent object for each noun. Valleau et al. recorded accompanying auditory stimuli and arranged the stimuli into the trial structure depicted in Figure 1. Experiment 1 included a subset of ten of the verb trials from the stimuli used by Valleau et al., described below, including only one item from each pair (e.g., “clap” but not “stretch”), as well as four of the noun trials which served as fillers to break up the session. All participants saw the same 14 trials in the same order.

#### Visual stimuli

Verb trials featured two dynamic scenes side-by-side. Eight verb trials featured dynamic scenes with an actor and an object (e.g., “shaking” and “opening” a present) and two trials featured dynamic scenes with just an actor (e.g., “clapping” and “stretching”). Within each trial, the actor and object were the same in both dynamic scenes (e.g., in the trial depicting “tickle” and “kiss,” one scene depicted a girl tickling a teddy bear, while the other depicted the same girl kissing the same teddy bear). Videos were looped to provide continuous depictions of the events. Filler trials targeting nouns featured two static images side-by-side.

#### Auditory stimuli

A female American English speaker recorded the auditory stimuli in a sound-attenuated booth. Children heard attention-grabbing phrases (e.g., “Wow!”) and directives to find the target. For trials including both an actor and object, verbs were targeted using transitive syntax (e.g., “Where is she tickling the bear?”), whereas those including only an actor were targeted using intransitive syntax (e.g., “Where is she clapping?”). Children also heard prompts in neutral syntax (e.g., “Find clapping!”).

### Design

Each trial included an Inspection Phase, a Baseline Phase, a Prompt Phase, and a Test Phase. See Figure 1. Verb trials and noun filler trials were structured identically; however, the Inspection and Baseline Phases were shorter for noun trials than for verb trials because static images do not change over time and we did not want children to tire of looking at them.

In the Inspection Phase (8 s for verb trials; 4 s for noun trials), children previewed each visual stimulus individually, one on the left and the other on the right. Side (left or right first) and order (target or distractor first) were counterbalanced. The Baseline Phase (6 s for verb trials; 3 s for noun trials) depicted both visual stimuli simultaneously in the same locations they had appeared in during the Inspection Phase. The Inspection and Baseline Phases included attention-grabbing phrases to direct children’s attention to the screen (e.g., “Look!”, “Wow!”).

In the Prompt Phase (4 s for verb and noun trials), children heard a prompt to find the target scene or image (e.g., “Where is she clapping?”). A centrally positioned star directed children’s attention to the center of the screen. In the Test Phase (6 s for verb and noun trials), the visual stimuli reappeared in their original positions. Children heard an additional prompt (e.g., “Find clapping!”).

### Procedure overview

Participation was part of a two-visit protocol approved by Boston University’s Institutional Review Board. At the first visit, parents provided written consent and completed a demographics questionnaire, the MBCDI and M-CHAT-R. The first author, a licensed speech-language pathologist, administered the PLS. Children also participated in an unrelated experimental task. At the second visit, approximately two weeks later, children took part in two additional experimental tasks, of which this study was the second. The MSEL-VR was also administered during the second visit.

### Exclusionary criteria

All trials with more than 50% track loss (e.g., blinks) during the Test Phase were removed from analysis. After these removals, on average, 9 of 10 verb trials (*SD* = 1, range = 5–10) were included for typically developing children, while 7.5 of 10 (*SD* = 2, range = 4–10) were included for late talkers; this difference was significant (*t*(43) = 3.35, *p* = .002). Differences in the number of included trials is unsurprising given that late talkers show differences in attention during experimental tasks (62). However, some of this inattentiveness may also be driven by task difficulty; for example, late talkers may look toward a parent or examiner for cues because they are unsure of the target word’s meaning.

### Analysis

Our analyses considered children’s 1) accuracy and 2) processing speed. For each, we conducted a mixed-effects regression to determine whether there were group differences.^2^ This included the outcome variable of eye gaze behavior (accuracy or processing); random effects of participant and trial; and fixed effects of age, gender, and group (late talker or typically developing). Regressions were run using the lme4 package (Version 1.1–12; 63) in R (64) with model comparisons made using the drop1() function with chi-square tests.

#### Accuracy

Following Reznick (7), we calculated accuracy as an increase of 15% in target looking between Baseline (before children are prompted to find the target) and Test (after the auditory prompt). To identify at what point in time during the 6-s Test window we should make this calculation, we applied a bootstrapped cluster-based permutation analysis (50) using the eyetrackingR Package (65). We hypothesized that late talkers might require a later time window for demonstrating vocabulary knowledge than typically developing toddlers, so we ran separate analyses for each group. The cluster analysis compared children’s gaze behaviors between Baseline and Test to identify if and when children preferred the target in the Test Phase above and beyond Baseline looking rates. For the Baseline phase, we averaged proportion of looks to the target scene versus elsewhere across all time points and trials to obtain a single measure of each group’s overall preference for the target scene during this Phase. This is because we were not interested in the dynamics of their attention to the target scene during Baseline, but rather how much they preferred to look at it overall. For the Test Phase, in which we were interested in the dynamics of children’s attention over time, we calculated children’s average proportion of looks to the target scene versus elsewhere in each 50-ms window. In both cases, when calculating the proportion of looking to the target, we included looks to neither the target nor the distractor (e.g., looking in between the two scenes) and track loss in the denominator of the proportion; these looks may reflect children’s uncertainty and we did not want to remove these data points.

Our planned model for identifying clusters was a mixed-effects regression with the dependent variable of proportion of looks to the target scene versus elsewhere, the predictor variable of phase (Baseline or Test), and random effects of trial and participant. We applied a threshold of *p* = 0.05, meaning the time bin had to reach this level of statistical significance in order to be included in a cluster. Adjacent clusters and those separated by only 50 ms were combined into larger clusters. We then ran the permutation analysis with 1000 permutations to confirm that these windows emerge even when the data is scrambled. A paired *t-*test (following, e.g., 66) compared, by child by trial, the average proportion of looks to the target scene between Baseline and the identified cluster.

The earliest statistically significant cluster was used to identify the response window for the accuracy analysis. Response windows—separate for each group—began at the start of the earliest significant cluster wherein children looked more to the target scene in the Test Phase than in Baseline. We standardized the duration of the response windows to 1500 ms, as has been done in receptive noun vocabulary tasks (e.g., 8).

Accuracy was then calculated, by-child by-trial, by comparing the average proportion of looks during the whole 6 s of the Baseline Phase and the response window of the Test Phase. A child was credited with knowing the meaning of the target verb if their looks increased at least 15% from Baseline to Test.

#### Processing speed

Processing speed was operationalized as latency, i.e., the earliest time point within the test phase of each trial in which the child looked toward the target scene. As in Valleau et al. (11), children who did not look to the target scene during the test phase at all were given a latency of 6000 ms. Also following Valleau et al. (11), we excluded looks in the first 50 ms of the Test Phase as being too early to be attributable to hearing the auditory stimuli; it takes approximately 200 ms to program and launch a saccade (e.g., 67).

### Results

**Figure 2.**
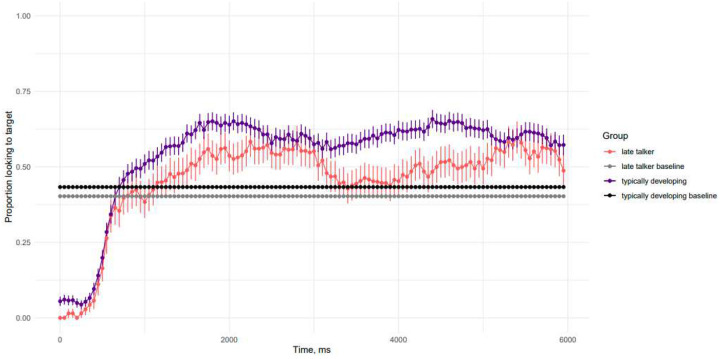
Timecourse of children’s gaze to the target scene during the test phase by group (Experiment 1). The x-axis represents time, in ms, from the onset of the test phase, and the y-axis represents the proportion of looks to the target scene versus elsewhere. Error bars indicate standard error of participant means. Dashed lines indicate group baseline averages.

De-identified gaze data are available on the Open Science Framework (https://osf.io/ghp7q). Figure 2 depicts children’s preference for the target scene over time as the Test Phase unfolded; target preference is calculated as the proportion of frames in which children looked to the target scene versus all other locations. Baseline looking preference is indicated by the dashed lines. Late talkers averaged a smaller proportion of looks to the target scene than typically developing children (*t*(43) = 3.8, *p* < .001). However, both groups preferred the target during the Test Phase above Baseline looking rates. This suggests that, overall, children know at least some of the target verbs queried.

#### Accuracy

For late talkers, the bootstrapped cluster-based permutation analysis revealed three clusters in which proportion looks to the target scene differed between Baseline looking rates (*P* = 0.40) and the Test Phase. The first cluster lasted from 0 to 600 ms: Here, late talkers looked less to the target scene in Test than they had in Baseline (*t*(104) = 15, *p* < .001). This is unsurprising given the trial structure: Recall that children begin the test phase looking at the center of the screen, as they have just seen a central fixation star. The second cluster began at 1550 ms and lasted to 3100 ms (*t*(104) = −2.5, *p* = .01); here, late talkers looked more to the target scene in test than in Baseline. The third cluster, from 4850 to 6000 ms, was not statistically significant (*t*(104) = −1.3, *p* = −.19, *n*.s.) after the permutation analysis and *t*-test. Given the results of this analysis, late talkers were given the response window of 1550 to 3050 ms for the accuracy analysis (recall that we standardized windows to a duration of 1500 ms).

For typically developing children, two significant clusters emerged in which gaze behaviors differed between Baseline (*P* = 0.43) and Test. The first cluster lasted from 0 to 600 ms. As with late talkers, typically developing children began the Test Phase looking less to the target scene than they had in Baseline (*t*(276) = 19, *p* < .001). The second cluster lasted from 900–6000 ms. Here, typically developing children looked at the target scene significantly more during test than they had during baseline (*t*(276) = − 5.7, *p* < .001). We therefore used a response window for the accuracy analysis of 900 to 2400 ms for typically developing children.

Using the threshold of 15% increase between Baseline and response window, late talkers knew 51% of the target verbs (*SD* = 0.22, range = 0.125 – 1) for trials they contributed. Typically developing children knew 49% of the target verbs (*SD* = 0.19; range = 0.0 – 0.9) for the trials they contributed. The regression model indicated no significant relationship between children’s accuracy and any of the fixed effects included (*b*_group_ = 0.02, *t*_group_ = 0.34, *p*_group_ = .70, *n.s*.; *b*_age_ = 0.02, *t*_age_ = 1.88, *p*_age_ = .054, *n.s*.; *b*_gender_ = −0.09, *t*_gender_ = −1.51, *p*_gender_ = .11, *n.s*.;). This indicates that, when provided enough time to demonstrate knowledge of target items, there are no significant differences in the number of verbs late talkers and typically developing children know.

#### Processing speed

Participants’ latency to look to the target scene averaged 1500 ms (*SD* = 502 ms). Surprisingly, late talkers (*M* = 1551 ms, *SD* = 477 ms) did not average longer latencies than typically developing children (*M* = 1477 ms, *SD* = 519 ms; *t*(43) = 0.66, *p* = .45, *n.s*.). The regression analysis indicated that age significantly predicted latency (*b* = −72, *t* = −4.9, *p* = .004), but group (*b* = −119, *t* = −0.75, *p* = .44, *n.s*.) and gender (*b* = 36, *t* = 0.25, *p* = .79, *n.s*.) did not.

### Discussion

In Experiment 1, we explored performance during a receptive verb vocabulary task with 2-year-old late talkers and typically developing children. We considered children’s overall accuracy and processing speed.

In calculating children’s accuracy, prior research has suggested that the response window that has been typically used with static images and noun stimuli (300 to 1800 ms) is inappropriate for dynamic scene targets (9, 11). One notable contribution of this work is that we identified a response window using bootstrapped cluster-based permutation analyses (50). Given that late talkers are slower lexical processors as compared to typically developing toddlers (20), it is perhaps unsurprising that they required a later window than their typically developing peers to demonstrate verb knowledge. While typically developing children preferred the target scene above and beyond Baseline looking rates beginning at 900 ms in the test phase, late talkers did not do so until 1550 ms. These findings echo research on older children with developmental language disorder, who show delayed responses during receptive language tasks (e.g., 68). However, when provided additional time, late talkers knew as many verbs as did typically developing children.

While not what we had hypothesized, this result is not altogether unsurprising. Late talkers are defined by the size of their expressive vocabularies; Prior research on receptive vocabulary indicates that some late talkers do not have deficits in this area (33). We also acknowledge that although late talkers and typically developing children knew on average the same number of verbs, it is not necessarily the case that they have equally robust representations of those verbs. Indeed, we observed in our bootstrapped cluster-based permutation analysis that, unlike typically developing toddlers, late talkers did not sustain a preference for the target scene once they identified it. This may be an indication that their representations are more fragile. How to operationalize robustness of a lexical entry remains an open question in the field, although some have proposed that overall looking time to the target may be one measure (69). We advocate for continued research into how best to operationalize robustness of representation.

Latency is a well-established eye gaze measure for processing speed given static images and noun targets, but research with dynamic scene stimuli has drawn mixed conclusions (10–11, 16). Although late talkers average slower latencies than typically developing children given noun targets and static images (20), we found no group differences in average latency to verb targets and dynamic scenes. Instead, children’s age significantly predicted performance, with older children faster to orient to the target than younger children. These results may provide insight into the discrepancies of prior findings. Golinkoff et al. (10) and Valleau et al. (11), who found no relationship between language ability and latency, both studied children who were younger than 2 years of age. However, Koenig et al. (16) did find that language predicted latency in 3-year-olds. We hypothesize that children are refining their processing abilities during the third year of life. Fernald et al. (70) observed that, given noun targets, typically developing children refine word-level processing between 15 and 24 months of age, advancing from whole-word to incremental processing of words. We propose that between ages 2 and 3, children continue to improve their incremental processing, which results in processing speed better reflecting other aspects of language knowledge.

## Experiment 2: Autistic preschoolers

### Participants

The final sample comprised 20 children (3 female, 17 male) on the autism spectrum who were recruited from the greater Boston area. Children averaged 41.9 months old at the time of participation (*SD* = 10.2, range = 26.5 to 64.5 months). Per parent report, all children had a diagnosis of autism spectrum disorder, autism, or PDD-NOS; diagnosis was confirmed in the lab with the ADOS-2 (Autism Diagnostic Observation Schedule, Modules 1–3 or Toddler Module; 71–72). Parents reported that their child was exposed to English at least 80% of the time and had no history of hearing loss or comorbid developmental disorders. Participants were white (90%) or mixed ethnicity (10%). The majority of mothers (65%) had at least a college degree; one family did not provide maternal education information. An additional 12 children participated but were excluded from analysis because they contributed insufficient data (see below).

Expressive vocabulary was assessed using the MBCDI Level 2 Short Form A (58). Parents reported that their children produced, on average, 62 of the 100 words on the checklist (*SD* = 32, range = 0–99). Parents of two children did not complete the MBCDI. Three subtests from the Mullen Scales of Early Learning (MSEL: 60), widely used with autistic children (e.g., 42), were administered. We report these scores as raw numbers with age equivalents because standardized scores may fail to capture variability within a narrowed range (73–74). On the Expressive Language subscale (MSEL-EL), the average raw score was 27 (*SD* = 9.4, range = 10–48, age equivalent = 29 months); on the Receptive Language subscale (MSEL-RL), 30 (*SD* = 9.5, range = 10–48, age equivalent = 33 months); and on the Visual Reception subscale (MSEL-VR), 32 (*SD* = 9.4, range = 12–50, age equivalent = 31 months). More than half the children’s *t-*scores were more than one standard deviation below the mean for their chronological age on the MSEL-RL (52%), and the MSEL-EL (57%), indicating that they had delayed language development. See Table 2. Children were randomly assigned to one of two stimuli lists described below; no group differences existed between lists with respect to age, MBCDI, or MSEL scores.

### Apparatus

Identical to Experiment 1.

### Stimuli

Whereas Experiment 1 used a subset of the stimuli developed by Konishi et al. (61) and Valleau et al. (11), in Experiment 2 we used all 36 verb trials and 14 filler noun trials. Otherwise, the stimuli and trial structure were identical to Experiment 1.

Children were randomly assigned to one of two trial lists (*N* = 10 children in each). Each list included the same visual stimuli, but the verb queried from the pair differed between the two lists. For example, on the trial depicting the events “clap” and “stretch,” children assigned to list 1 were asked to find “clap” while children assigned to list 2 were asked to find “stretch.” The order of trials varied between the lists. All children saw 18 verb trials and 7 filler noun trials, once each. Fourteen verb trials featured dynamic scenes with an actor and an object (e.g., “shaking” and “opening” a present) and four trials featured dynamic scenes with just an actor (e.g., “clapping” and “stretching”).

### Procedure overview

Participation was part of a two-visit protocol approved by Boston University’s Institutional Review Board. At the first visit, parents provided written consent and completed a demographics questionnaire and the MBCDI. Children were assessed on the three relevant subscales of the MSEL and participated in an unrelated experimental task. At the second visit, approximately one month later, children participated in this experimental task. The ADOS-2 was completed at the second visit or within a 6-month period of the child’s participation in this study.

### Exclusionary criteria

Given that autistic children, including those in our sample, vary widely in language abilities, we ran bootstrapped cluster-based permutation analyses separately for each child. This necessitated more stringent exclusionary criteria; we removed from analysis all trials in which track loss was greater than 33% during the Test Phase. We also removed from the sample all participants who lost more than half of their trials to this criterion (*n* = 12 of the original 32 participants). There were no significant differences in average age or in MBCDI or MSEL scores between excluded participants and those in the final sample. From the final sample of participants (*N* = 20), 17% of trials were excluded from analyses due to track loss.

### Analysis

As with Experiment 1, we were interested in two different measures: accuracy and processing speed. To analyze whether individual factors predicted eye gaze behaviors, we ran two mixed-effect regressions to explore the contributions of 1) vocabulary (MBCDI) and 2) receptive language (MSEL-RL). We elected for two models rather than one because MBCDI and MSEL-RL were highly correlated (*r* = 0.84). Each trial for each participant was included as a separate data point. Models included gaze behavior as the dependent variable (accuracy or processing speed), the random effects of participant and trial, and the fixed effects of language measures (MBCDI or MSEL-VR), age, gender, and MSEL-VR score.

#### Accuracy

Given the heterogeneity of language abilities for autistic children, we created individualized response windows for each child using a procedure similar to Experiment 1. First, we ran cluster-based analyses for each child using the eyetrackingR package (65) in R (Version 3.3.1; 64). Clusters were identified using a linear mixed-effects regression, which compared the child’s Baseline proportion of looks to the target scene, collapsed over the 6-second Baseline Phase, to their proportion of looks to the target scene in each 50-ms bin of the Test Phase. The model included the dependent variable of proportion looks to the target scene versus elsewhere, the random effect of trial, and the predictor variable of phase (Baseline or Test). Time bins with a *p*-value of less than 0.05 were included in a cluster. With Experiment 1, we then assessed whether clusters that emerged from this analysis were statistically significant using both a permutation analysis and, then, a paired *t-*test comparing Baseline to Test to determine a *p*-value for the cluster. However, given the small number of trials that each child contributed, we did not have enough power to run the necessary *t*-test; no child had more than 18 trials, and many had fewer. We acknowledge this as a limitation of this approach that will need to be addressed in subsequent research on the feasibility of individualizing response windows. Instead, we performed a paired *t*-test for all participants together and provide additional descriptive statistics on gaze behavior differences between Baseline and Test.

As with Experiment 1, adjacent time bins in which gaze behaviors differed significantly between Baseline and Test, and those separated by only 50 ms, were combined into a single cluster. The first cluster lasting at least 500 ms was termed the child’s “sustained preference window.” We required that the cluster lasted at least 500 ms to eliminate short clusters (e.g., 50–100 ms) that may have been spurious differences as a result of scanning behavior rather than an indication of vocabulary knowledge. Four children did not have any clusters lasting 500 ms, so their “sustained preference window” was instead their longest cluster.^3^ This sustained preference window was the basis for each child’s individual response window. Individual response windows began at the start of each child’s sustained preference window and were standardized to 1500 ms in duration (consistent with Experiment 1). Three children’s sustained preference windows began within the last 1500 ms of the test phase, so they were assigned the individual response window of 4500 – 6000 ms.

Accuracy was calculated by-child by-trial by comparing the proportion of looks to the target scene during the whole of the Baseline Phase and during the child’s individual response window of the Test Phase. Children were credited with knowing the verb if they demonstrated a 15% increase in looks between Baseline and their individual response window (7). Each child’s overall accuracy was calculated as a proportion of the number of trials correct over the number of trials contributed to account for the trials removed due to track loss.

#### Processing speed

As with Experiment 1 (and 11), latency was operationalized as the first look to the target scene, by-child by-trial. Looks during the first 50 ms were excluded as chance looking based on trial design, and participants who did not look to the target scene during the test phase were given a latency of 6000 ms.

### Results

**Figure 3.A – 3.C.**
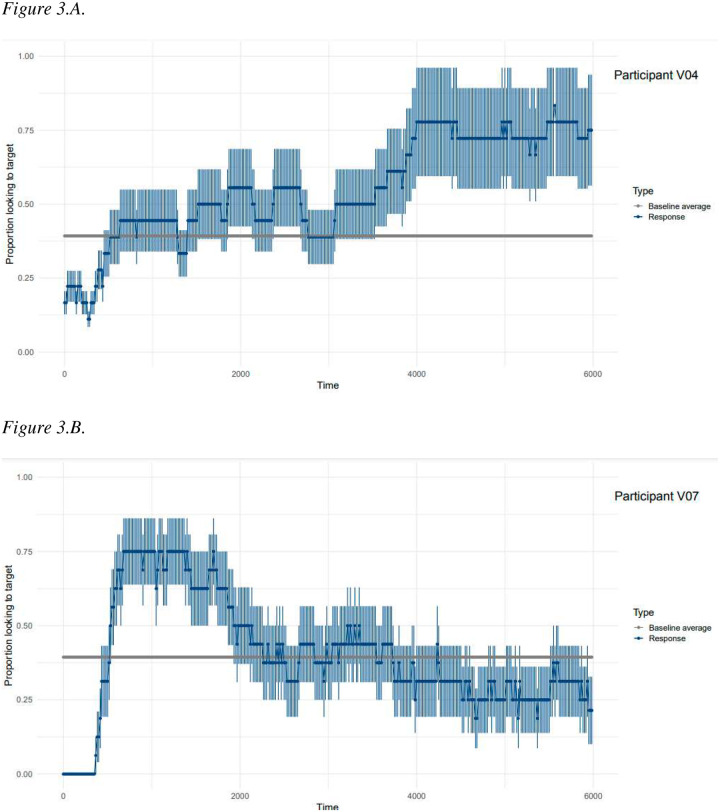

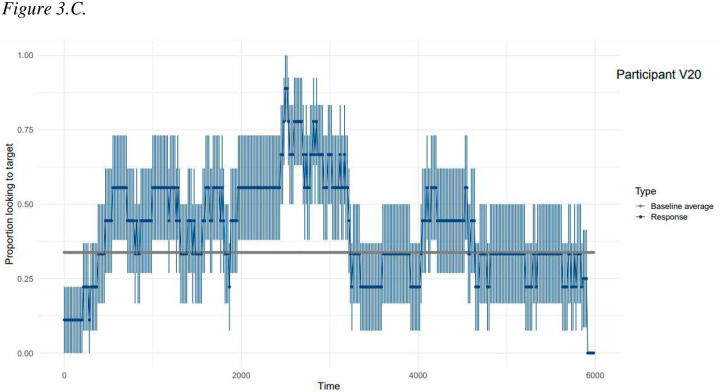
Timecourse graphs for three individual participants in Experiment 2. The timecourse displays each child’s average proportion of looks to the target scene versus elsewhere across the testphase. Error bars represent standard error of trial means. The horizontal bar represents children’s individual baseline average, calculated as the proportion of looks to the target versus elsewhere across the entire baseline phase, collapsing across all trials. Participants V04 and V07 had identical MBCDI and MSEL-RL raw scores. Participant V20 had the highest MBCDI and MSEL raw scores.

De-identified gaze data are available on the Open Science Framework (https://osf.io/egwya/). We began by graphing each participant’s individual performance. See examples in Figure 3. Here, children’s average proportion of looks to the target scene versus elsewhere (including looks to neither scene and track loss) during the Test Phase is depicted. The bar indicates the average proportion of looks to the target scene during all time points at Baseline. We observe that all three participants shown in Figure 3 preferred the target scene during Test above and beyond Baseline looking; however, each child did so at a different point. This is particularly striking for participants V04 and V07, who had identical MBCDI and MSEL-RL scores. Participant V07 demonstrated a preference for the target scene early in the Test Phase, whereas V04 did not do so until the latter half. V20, who had the highest scores of any participant on the MBCDI and MSEL-RL, looked most consistently to the target scene during the middle of the Test Phase.

#### Accuracy

Using cluster-based permutation analyses, we identified each child’s individual response window (See Table 3). As a group, the paired *t-*test indicated that average proportion of looks to the target scene significantly differed between the Baseline Phase (*M* = 0.41, *SD* = 0.21) and children’s individualized response windows in the Test Phase (*M* = 0.64, *SD* = 0.35; *t*(246) = 9.71, *p* < 0.001). However, there was considerable within-group variability for the average change in proportion looking between Baseline and the individualized response window (*M* = 0.22, *SD* = 0.12, range = −0.04 – 0.37).

Children knew, on average, 60% of the verbs queried (*SD* = 0.14, range = 0.40 – 0.83). In our vocabulary model, MBCDI scores had a marginal but non-significant relationship with children’s accuracy (*b* = 0.02, *t* = 1.86, *p* = .06, *n.s*.); no other factors were significant (*b*_age_ = −0.004, *t*_age_ = −0.89, *p*_age_ = .37, *n.s*.; *b*_gender_ = 0.03, *t*_gender_ = 0.27, *p*_gender_ = .78, *n.s*.; *b*_MSEL-VR_ = 0.008, *t*_MSEL-VR_ = 0.56, *p*_MSEL-VR_ = .57, *n.s*.). In our receptive language model, MSEL-RL scores predicted performance: Children with higher MSEL-RL scores performed better (*b* = 0.02, *t* = 2.18, *p* = .03). No other factors were significant (*b*_age_ = −0.004, *t*_age_ = −0.89, *p*_age_ = .37, *n.s*.; *b*_gender_ = 0.03, *t*_gender_ = 0.27, *p*_gender_ = .78, *n.s*.; *b*_MSEL-VR_ = 0.008, *t*_MSEL-VR_ = 0.56, *p*_MSEL-VR_ = .57, *n.s*.).^4^

#### Processing speed

Children averaged a latency of 1030 ms to look to the target scene (*SD* = 378, range 452–1761). In our vocabulary model, no fixed effect significantly predicted children’s latency to the target scene (*b*_MBCDI_ = −28, *t*_MBCDI_ = −0.79, *p*_MBCDI_ = .37, *n.s*.; *b*_age_ = −0.61, *t*_age_ = −0.04, *p*_age_ = .94, *n.s*.; *b*_gender_ = 101, *t*_gender_ = 0.22, *p*_gender_ = .81, *n.s*.; *b*_MSEL-VR_ = −43, *t*_MSEL-VR_ = −0.90, *p*_MSEL-VR_ = .31, *n.s*.). However, in our receptive language model, MSEL-RL scores significantly predicted latency (*b* = −109, *t* = −2.5, *p* = .008). No other factors were significant (*b*_age_ = 0.11, *t*_age_ = 0.08, *p*_age_ = .97, *n.s*.; *b*_gender_ = 13, *t*_gender_ = 0.04, *p*_gender_ = .99, *n.s*.; *b*_MSEL-VR_ = 6.4, *t*_MSEL-VR_ = 0.15, *p*_MSEL-VR_ = .85, *n.s*.).

We also explored possible correlations between latency and sustained preference window. These are distinct measures: Latency indicates the speed of children’s first look on any given trial to the target scene, but a sustained attention window reflects prolonged fixations on the target and is representative of children’s gaze behaviors across the whole of the experimental session. However, both may be indicative of children’s processing abilities. We found that children’s average latency across trials correlated with the start of the sustained preference window (*r* = 0.60, *t* = 2.92, *p* = .01), indicating that children with shorter average latencies were also faster to demonstrate a sustained preference to the target scene. Average latency also significantly negatively correlated with the length of the sustained preference window (*r* = −0.74, *t* = −4.33, *p* < .001), indicating that children with shorter average latencies also had longer sustained attention windows, perhaps suggesting more robust lexical representations.

### Discussion

In Experiment 2, we assessed the receptive verb vocabularies of autistic toddlers, considering both the number of words they know and how quickly they process language. We found that both children’s accuracy and processing speed were predicted by concurrent receptive language skills but not expressive vocabulary. This is likely due to the complex relationship between receptive and expressive language among autistic children. For example, our sample included two participants who were nonverbal (V01 and V03), one of whom (V03) knew nearly half of all verbs presented. We argue that this underscores the importance of full and accurate assessment of receptive language abilities.

One notable contribution of Experiment 2 is that we established response windows during the Test Phase that were individualized to each child’s gaze behaviors. This is an approach worth future research: Given the heterogeneity of profiles of autistic children (41–43), researchers and clinicians would be well served to have a systematic method of identifying the correct response window at the individual level. But, we also acknowledge major limitations to the approach we have presented. First, our study included too few trials overall, such that we were unable to determine whether the windows identified by the bootstrapped cluster-based permutation analysis for each child significantly differed from chance levels; our paired *t*-test was run at the group, rather than individual, level. A longer study with more trials, however, would likely have exceeded children’s attention spans. Thus, there is no easy resolution to this limitation. Second, we applied stringent exclusionary criteria—which was necessary to have enough data to run this bootstrap analysis—but in so doing excluded 12 of the initial 32 participants recruited for this study. This is sizeable. Although we engaged more children in our task than may be able to complete standardized assessments—for example, Brady and colleagues found that nearly half of minimally verbal 4- to 7-year-old autistic children are unable to pass screening items for the Peabody Picture Vocabulary Test (3)—this task with minimal task demands may still leave out many children.

## General discussion

Although prior research has demonstrated the feasibility of using eye tracking to assess receptive vocabulary of a variety of word types (e.g., 8–11), several gaps in the current literature must be addressed before this technology can be used in clinical settings. First, prior research has primarily focused on noun vocabulary depicted by static images. However, it is important to also assess verb vocabulary, which is more appropriately depicted with dynamic scenes. Second, most prior research has involved typically developing toddlers, although there are notable exceptions (e.g., 3, 20, 23, 26–28). However, children with language delays and disorders, including late talkers (Experiment 1) and autistic children (Experiment 2) may exhibit different patterns of gaze behavior during eye-tracking assessments. It is important that research address both of these gaps, and this study is a first step in that process.

Experiments 1 and 2 demonstrated one approach to measuring accuracy and processing speed, with considerations for both the populations and stimuli included. Accuracy—taken as evidence that a child knows a word—has been operationalized in previous work as a 15% increase in looking to the target from a baseline period to a “response window” that lasts from 300–1800 ms after the auditory prompt to look to the target. However, prior research has made clear that for dynamic scenes, a different response window is needed. We therefore applied bootstrapped cluster-based permutation analyses (50) to determine which portion of the Test Phase would be most appropriate.

In Experiment 1, we used bootstrapped cluster-based permutation analysis at the group level, hypothesizing that late talkers would require more time than their typically developing peers to demonstrate knowledge of target words. This hypothesis was confirmed: Late talkers required approximately one half-second more to orient their gaze to the target scene. Given this extra time, however, we observed no differences in the average number of verbs late talkers and typically developing children knew.

In Experiment 2, we applied bootstrapped cluster-based permutation analysis individually, yielding an individualized response window for each child in the final sample. This can be interpreted as evidence for the feasibility of the approach, but more research is needed to determine whether this is reliable and valid. Given the small number of trials included, our statistical power was limited and we were forced to run a group-level paired *t-*test. This is further exacerbated by the fact that permutation analyses assume exchangeability of data, but there is a higher interdependence of performance within a single participant, potentially leading to elevated Type 1 errors. Finally, we adopted conservative exclusionary criteria, losing a sizeable portion of the initial sample in the process. Future research must balance the needs for high quality data with the goal of including as many participants as possible.

A second commonly used eye-gaze measure, latency, is well established for assessing lexical processing given noun targets and static images, but researchers have disagreed on its use given verb targets and dynamic scenes (e.g., 11, 20). The current experiments provide insight into prior, discrepant findings. In Experiment 1, we found that age but not language predicted processing speed for late talkers and typically developing children. We hypothesized from these results that between 2 and 3 years of age there is a maturation of processing ability that results in a tighter relation between children’s language knowledge and their ability to quickly look at a named target. This hypothesis is supported by the findings of Experiment 2: For older, autistic preschoolers, the majority of whom (*n* = 14 of 20) were greater than 3 years of age, processing speed was reliably predicted by concurrent receptive language abilities. In further support for our hypothesis, we noted that in Experiment 1, 2-year-olds’ average latency to look to the target was 1500 ms, while in Experiment 2, older autistic children averaged a much shorter latency of 1030 ms, even though the majority of these children had delayed language. We suggest that between 2 and 3, when processing skills are developing, the child’s age best predicts latency because age is a proxy for how far along the child is in refining these skills. After age 3, these skills have been refined sufficiently and performance is instead driven by linguistic knowledge.

### Limitations and cautionary tales

In addition to those already discussed, we note several additional limitations to this study. First, the samples in both experiments are not representative of contemporary U.S. demographics: Participants were disproportionately white, and most came from higher socioeconomic backgrounds. Both limit the generalizability of our findings. For example, we acknowledge that children from higher socioeconomic backgrounds may have different experiences with technology, which may have impacted their engagement with the task.

Second, we interpret our accuracy measurement with a word of caution. Results indicate that late talkers (Experiment 1) and autistic children (Experiment 2) have some knowledge of the target verbs. However, we cannot claim from this measure that our children with language delays and disorders have equally robust representations to those of their typically developing peers’ (Experiment 1) or even to one another (Experiment 2). Indeed, it is likely that there are differences across and within groups robustness, as has been observed in older children with developmental language disorder (e.g., 75–76). Some studies have attempted to operationalize other eye-gaze behaviors to measure robustness (e.g., Yu and Smith (69), who observed differences in overall looking time between “strong” and “weak” word learners), but these measures are not well-established. We see some evidence for other between-group differences in gaze behavior, including overall proportion looking to the target, but further research in this area is required.

Finally, it is clear from the participant’s highly varied performance in Experiment 2 that researchers and clinicians must be able to individualize response windows to each child’s unique patterns of gaze behavior (see Figures 3A–3C). However, we caution readers against settling too quickly on bootstrapped cluster-based permutation analyses as the best approach to take. Although we demonstrated the feasibility of using method to identify individualized response windows, there remain many questions and concerns regarding this application. For example, the statistical approach for identifying individualized response windows must be reproducible, but we are unable to assess reproducibility given the current data. Second, as we have discussed, using this method requires a delicate balancing act between a high number of trials and participant demands and between inclusionary/exclusionary criteria that is stringent enough to run the analysis but relaxed enough to include the broad spectrum of abilities. Finally, we recognize that in a continually evolving field, new and better methods may soon be developed.

### Future directions

One benefit of assessing receptive vocabulary using eye gaze is that performance on noun trials predicts later language and developmental outcomes (e.g., 48); in theory, such information may have a role in clinical decision-marking. Although verbs may be particularly powerful predictors of later language skills (12), whether eye gaze measures for receptive verb vocabulary can predict later language abilities is yet unstudied. We propose future work considering whether verb processing (latency, for older toddlers) and broader processing abilities (start time of individualized response window) predict later outcomes. We are particularly interested in the latter: Although we found no concurrent relationship between children’s sustained attention window (from which we derived individual response windows) and language abilities, it may be that the confluence of factors that result in these individualized differences may impact long-term vocabulary acquisition. For example, if the starting time of a sustained attention window is reflective of children’s abilities to integrate visual and linguistic information, we might expect that this would impact vocabulary acquisition: Children who are slower to process words they know may miss opportunities to acquire the meanings of unfamiliar words in the same sentence (e.g., 48, 77).

We would also like to see further research exploring individualized response windows. Beyond the questions we have already raised about the reliability and reproducibility of the method, it would also be beneficial to explore this approach in a variety of populations. As technology continues to evolve and be incorporated into clinical practice, it is vital that speech-language pathologists have the tools to accurately assess a broad spectrum of language abilities.

## Conclusion

This study addresses two notable gaps in prior literature on assessing receptive vocabulary using eye tracking. First, an assessment task should include many types of words, including verbs; however, most prior studies have included only noun targets depicted by static images (e.g., 8, 20; although, see 9–11). Second, although some research has been done on assessing receptive vocabulary in children with language delays and disorders (3, 20, 23, 26–28), more research is warranted on how best to interpret eye gaze behaviors as an indication of linguistic knowledge. We demonstrated one way to measure children’s accuracy and lexical processing of verbs, given considerations for the type of stimuli (dynamic scenes rather than static images) and populations (late talkers, autistic children). Our findings highlight the importance of adapting these measures at the group and even at the individual level to account for variation in performance.

In considering accuracy, we have demonstrated the feasibility of applying a bootstrapped cluster-based permutation analysis (50) to identify response windows for calculating accuracy. Using this approach, we found in Experiment 1 no differences in the average proportion of verbs that late talkers and typically developing toddlers knew, although late talkers required more time to demonstrate this knowledge. In Experiment 2, we found that the number of verbs children knew in the eye tracking task was predicted by their receptive language abilities. However, we interpret these results with caution: As highlighted in our discussion, we were underpowered to run *t*-tests for significance given individual windows, limiting our interpretations. We strongly encourage future research on this or alternative approaches to individualizing response windows based on gaze behavior.

In considering lexical processing, results from Experiments 1 and 2 shed light on prior, discrepant conclusions about the appropriateness of using latency as a measure of lexical processing given dynamic scene stimuli. We argue that latency is not a reliable measure of lexical processing for children younger than three years of age. In Experiment 1, age but not language ability predicted latency for two-year-old late talkers and typically developing children. In Experiment 2, however, receptive language skills predicted latency for older autistic preschoolers. Future research is warranted on how best to operationalize lexical processing for dynamic scene stimuli in younger children.

Using eye gaze may allow us to assess receptive vocabulary skills in children who might otherwise be unable to participate in standardized assessments (1–7). The findings of this study support this long-term goal, but more research is needed in how best to adapt assessments to such populations.

## Figures and Tables

**Table 1.** Late talkers’ and typically developing children’s performance on standardized assessments (Experiment 1).

	MBCDI	PLS-AC	PLS-EC	MSEL-VR
Late talkers	*M* = 40	*M* = 88	*M* = 90	*M* = 44
	*SD* = 25	*SD* = 15	*SD* = 11	*SD* = 10
Typically developing children	*M* = 82	*M* = 114	*M* = 114	*M* = 58
	*SD* = 16	*SD* = 12	*SD* = 12	*SD* = 8
Test statistic	*t* = 6.73,	*t* = 6.31	*t* = 6.17	*t* = 4.77
	*p* < 0.001	*p* < 0.001	*p* < 0.001	*p* < 0.001

**Table 2:** Participant characteristics (Experiment 2). Participants V02 and V03 and participants V10 and V19 are sets of twins.

ID	Age	Sex	List	MBCDI	Mullen Scales of Early Learning (raw scores)		
					Visual Reception	Receptive Language	Expressive Language
V01	26.5	M	1	0	12	10	10
V02	26.8	M	1	20	21	11	14
V03	26.8	M	1	0	24	16	14
V04	33.6	M	1	85	39	34	35
V05	40.3	M	1	31	31	27	16
V06	42.9	M	1	74	32	31	40
V07	44.6	M	1	85	32	34	32
V08	44.7	M	1	80	45	41	30
V09	48.1	M	1	75	45	31	28
V10	56.1	F	1	54	29	27	27
V11	33.7	M	2	77	30	36	31
V12	35.5	F	2	89	21	34	35
V13	36.8	M	2	63	40	30	27
V14	40.4	M	2	29	.	.	.
V15	41.5	M	2	.	28	26	18
V16	42.4	M	2	67	31	28	29
V17	45.3	M	2	97	44	40	40
V18	51.1	M	2	.	26	30	31
V19	56.1	F	2	87	34	32	30
V20	64.6	M	2	99	50	48	48

**Table 3:** Children’s sustained preference window, individual response window, and accuracy proportions (Experiment 2).

ID	Sustained Preference Window	Individual Response Window (for accuracy analysis)	Accuracy proportion (from individual response window)
V01	.	.	.
V02	1600 – 1900 ms	1600 – 3100 ms	0.44
V03	3300 – 3900 ms	3300 – 4800 ms	0.40
V04	3850 – 6000 ms	3850 – 5350 ms	0.83
V05	.	.	.
V06	800 – 2300 ms	800 – 2300 ms	0.67
V07	550 – 1750 ms	550 – 2050 ms	0.75
V08	1150 – 3000 ms	1150 – 2650 ms	0.69
V09	4700 – 5100 ms	4500 – 6000 ms	0.50
V10	3900 – 4700 ms	2900 – 5400 ms	0.50
V11	1400 – 2750 ms	1400 – 2900 ms	0.75
V12	4700 – 6000 ms	4500 – 6000 ms	0.67
V13	400 – 3050 ms	400 – 2100 ms	0.67
V14	5800 – 5950 ms	4500 – 6000 ms	0.46
V15	1600 – 1650 ms	1600 – 3100 ms	0.42
V16	.	.	.
V17	800 – 2600 ms	800 – 2300 ms	0.82
V18	500 – 1400 ms	500 – 2000 ms	0.63
V19	2450 – 3850 ms	2450 – 3950 ms	0.64
V20	2450 – 3200 ms	2450 – 3950 ms	0.44

## Data Availability

De-identified data are publicly available on the Open Science Framework (Experiment 1: (https://osf.io/ghp7q/; Experiment 2: https://osf.io/egwya/).

## Footnotes

1 Only two participants in Experiment 1 did not have at least one parent with a college degree.

2 We additionally ran exploratory analyses in which language was treated as a continuous variable. However, we found no significant effect of the language variables (MBCDI raw score; PLS-AC standard score).

3 Three children did not have any significant 50-ms time bins (V01, V05, V16). These participants were excluded from further accuracy analyses.

4 As a post-hoc analysis, we explored whether the start or duration of children’s sustained preference windows correlated with individual factors. We found that the duration of the sustained preference window correlated with MBCDI scores (*r* = 0.60, *t* = 2.7, *p* = .02), but not MSEL-RL scores (*r* = 0.32, *t* = 1.3, *p* = .23, *n.s*.) or age (*r* = −0.02, *t* = −0.08, *p* = .94, *n.s*.). This suggests that children with larger vocabularies can direct more sustained attention to a labeled scene. The starting time point of children’s sustained preference windows did not correlate with language abilities (*r*_MBCDI_ = −0.25, *t*_MBCDI_ = −0.97, *p*_MBCDI_ = .35, *n.s*.; *r*_MSEL-RL_ = 0.03, *t*_MSEL-RL_ = 0.10, *p*_MSEL-RL_ = .92, *n.s*.) or age (*r* = −0.03, *t* = −0.13, *p* = .90, *n.s*.), indicating that how early children settle on the target scene is independent of language or broader developmental factors.
